# Investigation of the Pd Nanoparticles-Assisted Chemical Etching of Silicon for Ethanol Solution Electrooxidation

**DOI:** 10.3390/mi10120872

**Published:** 2019-12-12

**Authors:** Olga Volovlikova, Gennady Silakov, Sergey Gavrilov, Tomasz Maniecki, Alexander Dudin

**Affiliations:** 1 Institute of Advanced Materials and Technologies, National Research University of Electronic Technology (MIET), Moscow 124498, Russia; mr.komrad-13@ya.ru (G.S.); pcfme@miee.ru (S.G.); 2 Institute of General and Ecological Chemistry, Lodz University of Technology, Zeromskiego 116, 90–924 Lodz, Poland; tmanieck@p.lodz.pl; 3 Institute of Nanotechnology of Microelectronics of the Russian Academy of Sciences (INME RAS), Moscow115487, Russia; dudin_aa@icloud.com

**Keywords:** porous silicon, Pd nanoparticles-assisted chemical etching, etching rate, ethanol electrooxidation

## Abstract

The formation of porous silicon by Pd nanoparticles-assisted chemical etching of single-crystal Si with resistivity *ρ* = 0.01 Ω·cm at 25 °C, 50 °C and 75 °C in HF/H_2_O_2_/H_2_O solution was studied. Porous layers of silicon were studied by optical and scanning electron microscopy, and gravimetric analysis. It is shown that por-Si, formed by Pd nanoparticles-assisted chemical etching, has the property of ethanol electrooxidation. The chromatographic analysis of ethanol electrooxidation products on por-Si/Pd shows that the main products are CO_2_, CH_4_, H_2_, CO, O_2_, acetaldehyde (CHO)^+^, methanol and water vapor. The mass activity of the por-Si/Pd system was investigated by measuring the short-circuit current in ethanol solutions. The influence of the thickness of porous silicon and wafer on the mass activity and the charge measured during ethanol electrooxidation was established. Additionally, the mechanism of charge transport during ethanol electrooxidation was established.

## 1. Introduction

Technological innovation leads to an increase in energy and natural resources consumption, in particular, natural gas. Continuous consumption of non-renewable resources leads to their exhaustion. There is a need to develop and make use of alternative energy sources, based on environmentally-friendly technologies, because of natural resources limitations. Development of new alternative energy sources will allow us to receive scientific and technical results, with technologies which provide transition to resource-saving energy. One of the prospective directions in the resource-saving power area is the fuel element and portable electrochemical energy generators. They have many advantages: portability, high efficiency, small level of harmful emissions and quietness [1]. Nowadays, an interesting and prospective direction in the resource-saving power area is ethanol as fuel and a power source for the electric current generators [2].

Transformation to electric power is due to direct ethanol oxidation in the cell. It allows the simplification of the fuel supply system because of high specific energy of liquid alcohols, providing a short circuit in an environmentally-friendly cycle of transformation of energy in the natural scale due to a number of alcohols. The ethanol can be produced in biosystems in almost unlimited volumes [3]. The oxidation product of ethanol is CH_4_.

Recent studies for alternative fuels indicate a growing interest in the development of small fuel cells and energy generators based on porous silicon due to a few advantages: high specific surface; strong chemical loading ability of the surface; the possibility of changing the surface morphology of the porous layers at the nano- and micro levels; simplicity and low cost of manufacture and compatibility with silicon integrated technology [4]. The plasma chemical etching for porous silicon formation is widely used. This method is characterised by a high complexity of hardware provided [5]. Chemical etching in solutions of alkalis or acids is a cheaper method for porous Si formation. Porous Si is usually formed by anode etching in HF solutions [6,7,8]. However, this method means individual treatment of wafers. 

In recent years, special development was received by the chemical etching induced by noble metals (Ag, Au, Pt, and Pd) [9,10,11,12]. This method is simple and enables carrying out group treatment of wafers that reduces the price of the technology of porous silicon formation. Besides, this method allows the production of porous silicon with a wide range of geometrical sizes by using a form and type of a metal mask. The thickness of the porous silicon is defined by the etching duration, electrolyte composition, and the metal amount. The MACE (metal-assisted chemical etching) method enables the production of a noble metal/porous silicon structure that combines the functions of both the anode and cathode of the generator’s active element and fuel cell. This structure is called the Schottky junction (Schottky barrier). In work by Bin Zhu [13,14] a hydrogen fuel cell based on the Schottky barrier (metal, *p*-type semiconductor) was described.

Porous silicon functionalised with noble metals is of undoubted scientific and practical interest as an object for the production of new energy generators. Simplifying the design by switching to a single-layer functional structure will increase the productivity and reduce the cost of the finished device. Conducting studies of the electrocatalytic ethanol oxidation using the cathode/anode structure, based on Pd clusters in a porous layer, allows producing an environmentally-friendly and resource-saving energy sector. The efficiency of creating a structure is associated with a high specific surface area of the porous silicon, and size effects of a metal catalyst during the ethanol electrooxidation. It occurs on the local nano- and micro-regions of the anodes/cathodes inside of porous silicon. Understanding the electro-catalytic activities of the ethanol on the Pd/por-Si structure is very important for developing more active catalysts for the direct-ethanol generators. The purpose of the work is establishment of the influence of the porous layer thickness and porosity formed by Pd nanoparticles-assisted etching on the duration of gas evolution, and ethanol electrooxidation mass activity for different ethanol-based electrolytes.

## 2. Materials and Methods

Boron doped *p*-type silicon wafers with (1 0 0) orientation and resistivity of 0.01 Ω·cm were cleaned as follows: (i) dipping into H_2_SO_4_ (98%):H_2_O_2_ (30%) (in volume 1:1) solution, then (ii) into HF (40%):H_2_O (in volume 1:4) solution to remove native oxide and finally, (iii) into pure ethanol. The cleaned wafers were cut into pieces 3 × 3 cm^2^. The samples were placed in a Teflon cell. The samples were immersed into solution PdCl_2_:HCl (0.5 g/L PdCl_2_, 20 mL/L HCl) for 30 min at 25 °C for Pd film deposition. The por-Si formation was performed in solution HF (40%):H_2_O_2_ (30%):H_2_O (25:10:4 in volume) at *T* = 25, 50 and 75 °C. Porosity was calculated by gravimetric analysis. Samples with area *S* were weighed before etching (*m*_1_). Then non-polished side of the sample was covered by varnish and dried in the air. The etching duration was 30–120 min. Then, samples were cleaned in ethanol and dried at 65 °C. The varnish was removed and the samples were weighed (*m*_2_). Por-Si was dissolved in a water solution of NaOH and weighed (*m*_3_). Porosity was calculated by the equation:

(1) $$P=\frac{m_{1}-m_{2}}{m_{1}-m_{3}}\times 100\%,$$

where *m*_1_ and *m*_2_ are the sample’s masses before and after etching and *m*_3_ is the sample mass after por-Si dissolution. Samples were etched in the same conditions because of the multi-sectional Teflon cell.

**Figure 1:**
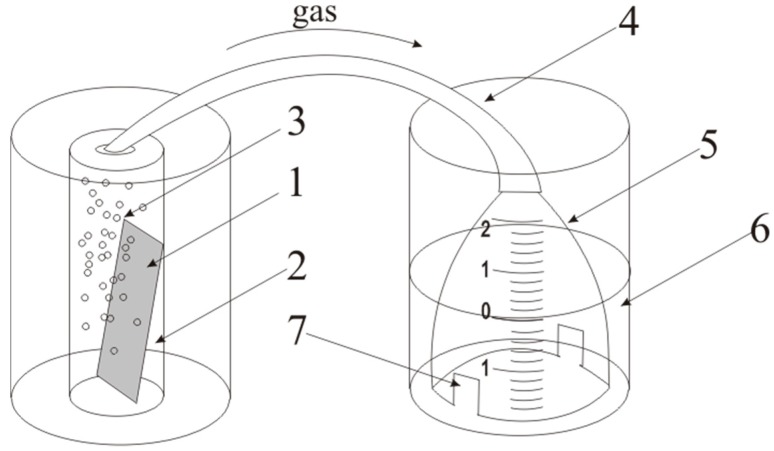
Schematic presentation of cell for gas volume measurement: (**1**) sample, (**2**) ethanol solution, (**3**) gas, (**4**) flexible tube, (**5**) cell, (**6**) glass cell, (**7**) holes.

The duration of filling of the same gas volume (2.4 mL) during ethanol electrooxidation (EEO), with different C_2_H_5_OH concentrations, was fixed. The gas volume, as a result of the ethanol electrooxidation by 2.5 cm^2^ samples, was measured using a cell (Figure 1). The essence of the method is the displacement of water by the gas, under a glass cell.

The sample (1) was placed into a glass cell with ethanol solution (2). Gas evolution (3) occurs. Then, gas follows on a flexible tube (4) in cell (5) located in glass cell (6) with water (level 0). Then the gas displaces water from cell through the holes (7) and the level changed. The displaced water volume equals to gas volume evolved during a certain time *V*_water_ = *V*_gas_.

The cell was filled multiple times to calculate the gas evolution rate *υ*. It was calculated by the equation:

(2) $$\upsilon =\frac{V_{\mathrm{gas}}}{t}$$

where *V*_gas_ is the gas volume and *t* is the duration of the gas evolution.

Sample surface morphology was investigated by optical and scanning electron microscopy (Carl Zeiss Axiovert 40 MAT (Carl Zeiss Group, Oberkochen, Germany) and Helios NanoLab 650 (Thermo Fisher Scientific, Hillsboro, OR, USA)). Energy-dispersive X-ray (EDX) spectroscopy was performed on FEI Helios (FEI, Hillsboro, OR, USA) with an EDAX Octane Elite super EDS System (Octane Super, Mahwah, NJ, USA). The short-circuit current during etching in the galvanic cells and the mass activity as a function of EEO time were measured with a digital multimeter (UNI-T UT61C, UNI-T Group Ltd., Hong Kong). The value of the charge *Q*_Excess Carrier_ was determined by numerical integration of current versus time, *Q* = $\int_{0}^{t}Jdt$. The composition and conductivity of ethanol solutions for mass activity measurements are presented in Table 1. pH equals to 2 made by adding H_2_SO_4_ into ethanol solution.

The gas composition was measured using quadrupole mass selective detector Hiden Analytical HPR-20 (Hiden Analytical, Warrington, UK) in the *m*/*z* range 1–100. EEO was performed in glass reactor at *T* = 25 °C, *p* = 1 bar. To obtain information on types of chemical bonds presented in the porous layer, the samples were analyzed by infrared (IR) reflectance spectroscopy using Nicolet iS50 spectrometer (Thermo Fisher Scientific).

## 3. Results

**Figure 2:**
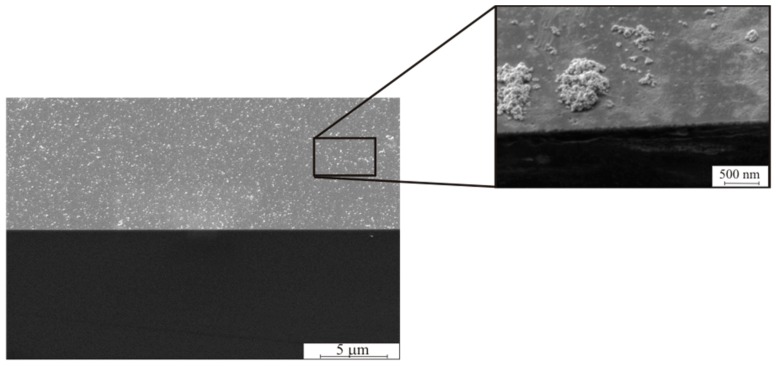
Scanning electron micrograph (SEM) image of Pd particles deposited on Si (1 0 0) wafer by immersing in PdCl_2_:HCl solution for *t* = 30 min.

Figure 2 shows scanning electron micrograph (SEM) images of Si (1 0 0) surface with palladium clusters deposited during immersion in PdCl_2_ solution for 30 min. The dimension of separate Pd particles varied in the range of 20 < *d* < 50 nm. The agglomerates from the Pd particles varied in the range of 0.1 < *d* < 2 μm.

**Figure 3:**
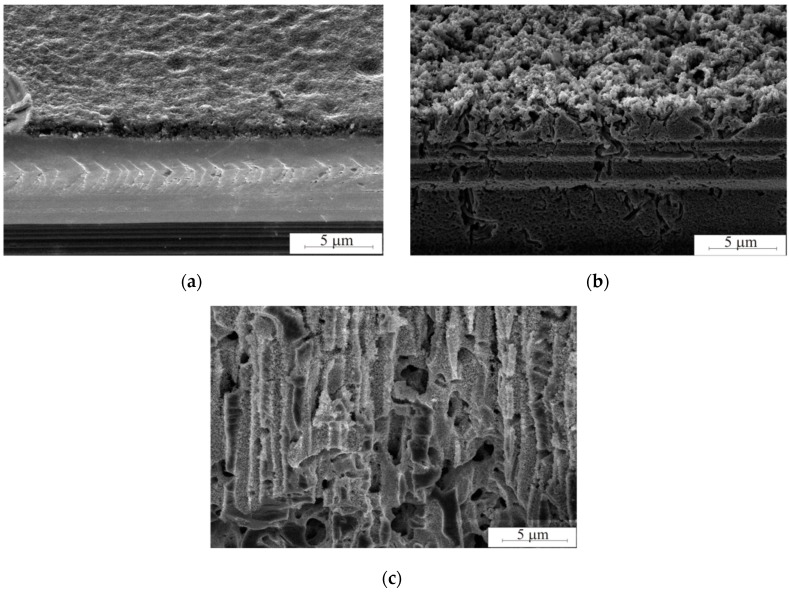
Scanning electron micrograph of the cross section porous silicon film etched for (**a**) 2 min, (**b**) 15 min and (**c**) 120 min at 25 °C.

Figure 3 shows SEM images of the porous silicon surface after Pd nanoparticles-assisted chemical etching with different etching times.

Figure 3 illustrates porous silicon formation during different etching times. The minimum etching duration was 2 min because of 1.25 μm porous layer is observed. The maximum etching duration was 120 min. The porous layer breaks after 120 min etching. The porous silicon has perpendicular macropores with diameters from 1 to 3 μm and mesopores tightly penetrating the walls of the porous matrix. Pd particles dissolve silicon, gradually plunging inwards and forming a pore. Parts of the particles were deposited on the pore walls during the etching process. This contributed to the formation of mesopores.

**Figure 4:**
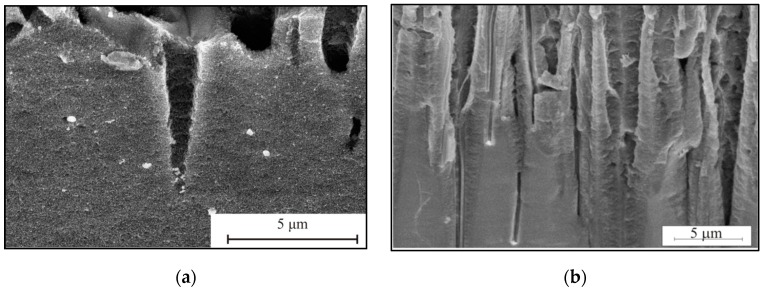
SEM image of cross section of sample after etching during 60 min: (**a**) the Pd particles are agglomerated, (**b**) the separate Pd particles.

Each pore is characterised by the size and shape of the metal particle. The appearance of the inner pore cavity is a consequence of the etching. If the particle is agglomerated, the pore walls are like a sponge of mesoporous silicon. As the agglomerate moves deeper into the pore, individual particles with a diameter of 80 ± 5 nm are deposited on the pore wall, contributing to its dissolution. This phenomenon is described in [12]. As particles are deposited on the walls, the agglomerate decreases in size, forming a dimple of a smaller diameter, which ultimately leads to the formation of a conical pore. In this case, the conical pore is due to the gradual dissolution of the walls (Figure 4a). Etching segments can determine the particle size. The upper part of the pore promotes its expansion with the solution penetrating the portion, uniformly dissolving the walls of the pore by photoelectrochemical dissolution. In the case of the separate metal particles, the pores will be formed vertically (Figure 4b). The pore diameter will be equal to the particle diameter.

**Figure 5:**
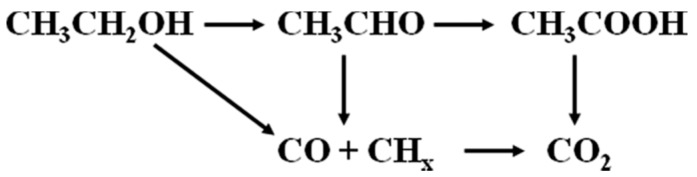
The scheme of the ethanol electrooxidation.

It was found that Pd particles are present in the mesopores, which have access to a system por-Si/Pd. This system provides the ethanol electrooxidation (EEO). It can proceed according to one of a few schemes (Figure 5) [15]. The acetaldehyde, acetic acid (incomplete oxidation) and carbon dioxide (complete oxidation) are the products of electrooxidation of ethanol [16].

**Figure 6:**
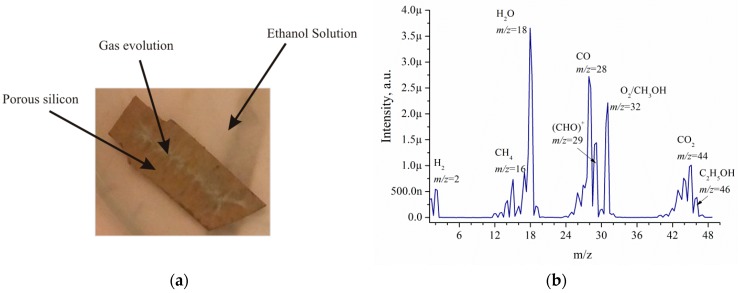
Electrooxidation (EEO): (**a**) sample photo during EEO, (**b**) the mass spectrum of a gas.

Figure 6a illustrates EEO. Intense gas evolution is observed from the functionalised metal-based silicon porous material immersed in ethanol solutions. Intense gas evolution gradually decreased. The gas evolution duration can be as long as several hours. The gas composition is shown in Figure 6b. 

**Figure 7:**
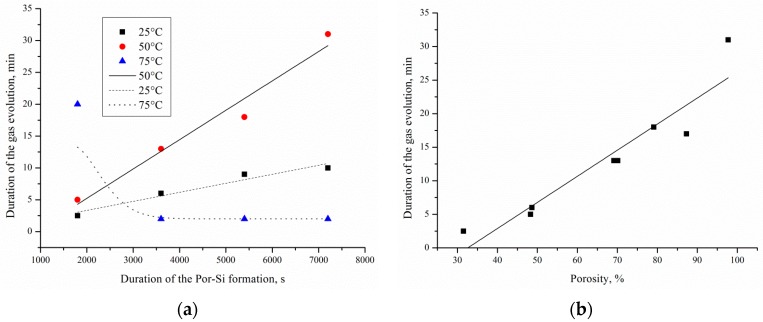
The dependence of the gas evolution on the (**a**) samples treatment duration, (**b**) por-Si porosity. Points are experimental results, lines are approximations.

The mass spectrometry analysis of EEO products on por-Si/Pd shows that the main products are CO_2_, CH_4_, H_2_, CO, O_2_, acetaldehyde (CHO)^+^ [17], methanol, ethanol and water vapor. The volumes of components relative to the total volume of EEO products are the following: H_2_—5%, CH_4_—7%, H_2_O—33%, CO—25%, O_2_—20%, CO_2_—10%. Figure 7a,b shows the influence of the sample porosity, etching duration and temperature on the duration of gas evolution. In this work, intensive gas evolution can be visually detected without using additional devices.

It has been found that the maximum duration of intense gas evolution (32 min) is observed for samples formed at 50 °C for 120 min etching, and the minimum duration (2 min) is observed for samples formed at 75 °C for 60–120 min etching. Increase and reduction of the duration of gas evolution is caused by the increase and reduction of porous silicon thickness respectively. It was established that a linear increase in the thickness of the porous layer happened with an increase in the etching duration from 30 to 120 min for temperatures of 25 and 50 °C. The thickness of the porous layer was from 30 to 90 μm for 30–120 min and 25 °C etching; from 60 to 105 μm for 30–120 min and 50 °C etching; and from 100 to 45 μm for 30–120 min and 75 °C etching. It is due to porous layer dissolution. Reducing the porous silicon thickness leads to a decrease of the local metal/semiconductor and the EEO regions, respectively. The transfer of porous silicon at elevated processing temperatures is described in detail in [18]. The duration of the gas evolution is linearly dependent on the porosity of the layer. Therefore, the high porosity of the sample ensures access of the reactants to the surface of por-Si/Pd and removal of the reaction products.

Besides the duration of gas evolution, an important parameter for establishing the EEO mechanism is the gas evolution rate. It characterises the EEO reaction rate. Table 2 shows the results of the analysis of the rate of gas evolution.

It has been found that the rate of gas evolution and the EEO is higher for solution 95/5. The high concentration of ethanol molecules promotes rapid adsorption. In addition, the rate of gas evolution is gradually reduced, which may be due to several factors:

1. solution depletion,
2. porous layer destruction,
3. contamination of the porous layer surface with reaction products.

The first two factors do not have an effect on the rate reduction. The porous layer destruction (SEM) and solution depletion has not been established. The addition of alcohol to the solution after the gas evolution stopped did not resume the process, while the as-prepared sample oxidized the spent solution. Treatment of the used sample in hydrofluoric acid contributed to the resumption of intense gas evolution in the spent solution.

**Figure 8:**
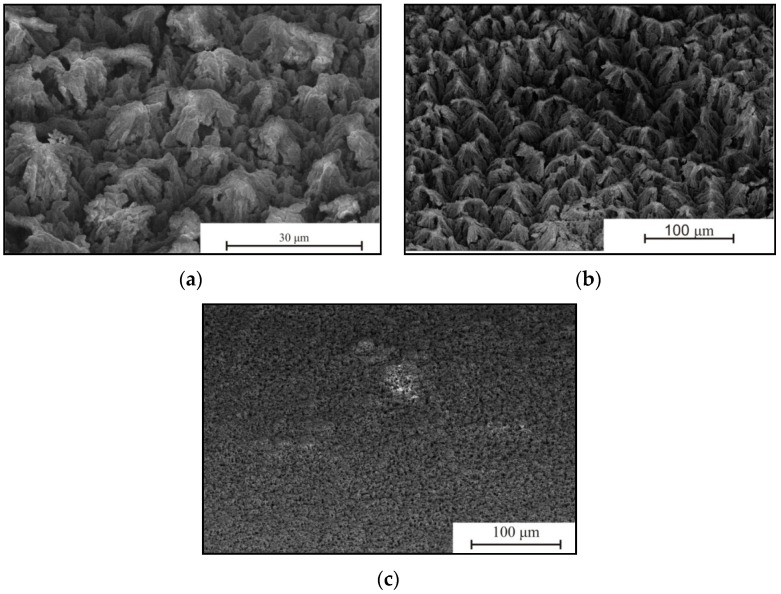
SEM images of porous silicon after EEO of solutions contained (**a**) 95/5, (**b**) 60/40 and (**c**) 30/70 ethanol/water at 25 °C during 30 min.

Figure 8 shows SEM images of Pd/por-Si surface after EEO.

**Figure 9:**
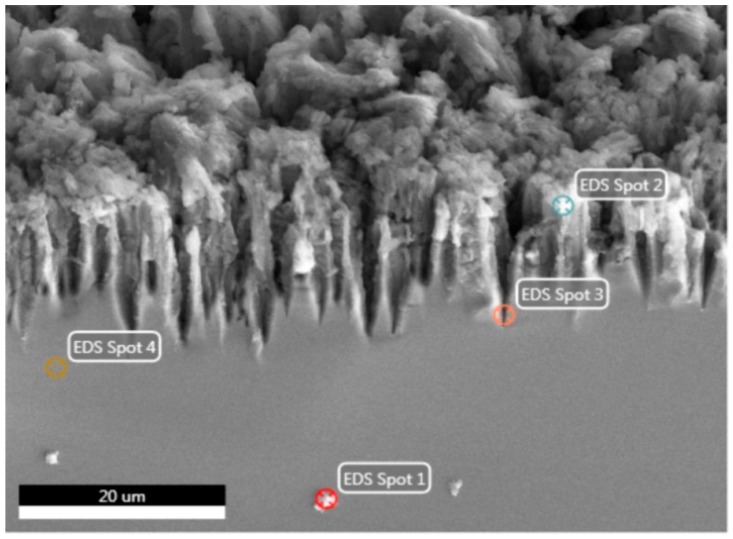
SEM image of porous silicon after electrooxidation and energy-dispersive X-ray (EDX) analysis spots.

In Figure 8, it can be seen that the gradual reduction of the gas evolution rate was due to contamination of the surface with reaction products. The higher the concentration of ethanol in the solution, the denser the precipitates. The thickness of the precipitate layer covering the porous layer has a value of several micrometres. The element analysis (EDX method) of the porous surface after EEO (Figure 9) allows us to determine a non-uniform distribution of elements into the surface. The elements in porous silicon are Si, O, and C (Table 3). The element in porous silicon at Spots 1, 3 and 4 is silicon.

**Figure 10:**
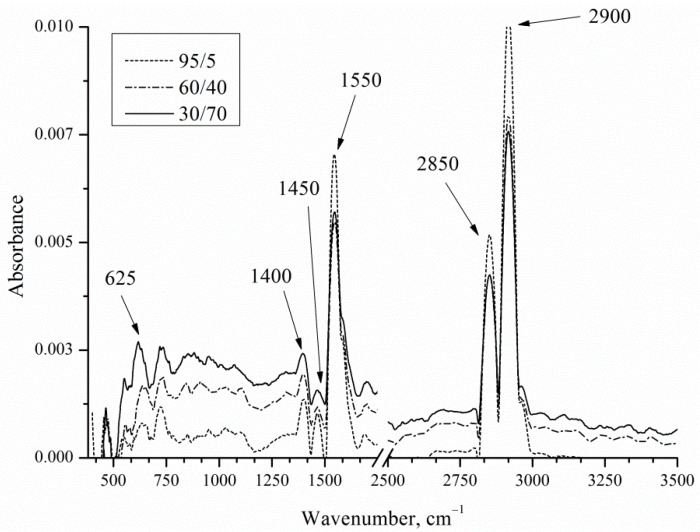
FTIR (Fourier-transform infrared) spectra of porous silicon after 120 min of electrooxidation of ethanol with different concentration.

Chemical bonds between the components of the porous layer were analyzed by infrared (IR) reflectance spectroscopy (Figure 10).

IR reflection spectra show the presence of bands typical for EEO by Pt and Pd catalysts. Table 4 presents wavenumbers corresponding to the bonds.

The CO_3_^2−^ may be observed near 2846 cm^−1^. The acetate was displayed as two intense peaks at 1553 and 1410 cm^−1^. As the concentration of ethanol in solution increases, the acetate of CH_3_COO^−^ band (1550 cm^−1^) and *ν* (C−H) of CH_3_CH_2_OH band (2900 cm^−1^) intensities also increase. As the concentration of ethanol in solution increases, the Si-H wag band intensities decrease. Decrease in intensity may be due to the increasing of the thickness of the precipitate layer covering the porous layer.

## 4. Discussion

The studies of EEO products on platinum catalysts using various analytical methods show that the reaction predominantly involves ethanol oxidation to CO_2_ [22]:

Anodic reaction on Pd:

(3) $$C_{2}H_{5}\mathrm{OH}\overset{2e}{\to }{\mathrm{CH}}_{3}\mathrm{OH}\overset{2e}{\to }{\mathrm{CH}}_{X}+\mathrm{CO}\overset{8e}{\to }2{\mathrm{CO}}_{2}$$

Cathodic reaction on Si:

(4) $$12H^{+}+3O_{2}+12e^{-}\to 6H_{2}O$$

**Figure 11:**
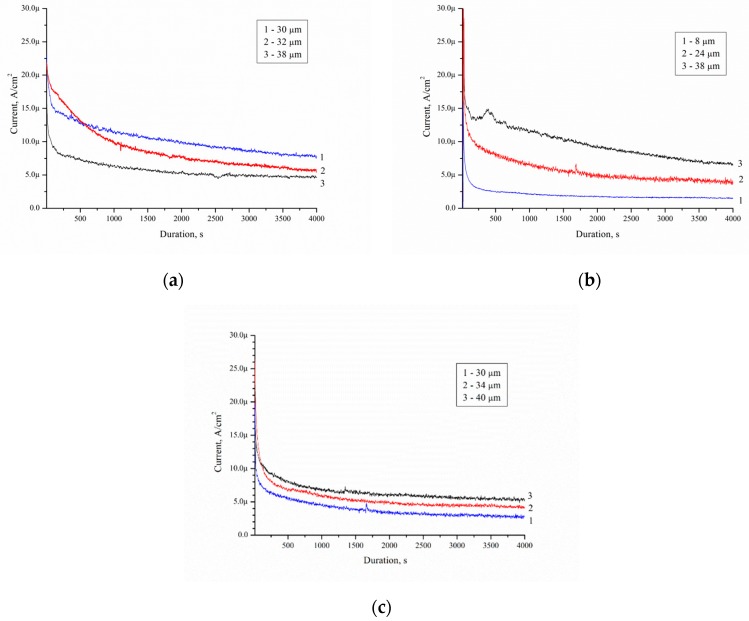
Current–time curves measured on Pd/por-Si for solutions: (**a**) No. 1 (10/90), (**b**) No. 2 (50/50) and (**c**) No. 3 (95/5).

O_2_ and H_2_ is a result of water splitting by porous silicon because of water solutions of ethanol [23]. Figure 11 shows the mass activity as a function of time on the Pd/porous silicon with a different thickness of por-Si for the solution No. 1–3. The mass activity characterises the amount of ethanol that was oxidized by the sample over a period of time *t* and the behavior of the process. The measurement was performed in a two-electrode cell, Pd/Si- anode, Pt- cathode. When the Si/Pd-system is dipped into the ethanol solution, EEO is occurred at the Pd/Si surface. The electrons’ transport is going through the porous layer and silicon wafer. The electrons diffuse into the semiconductor and accumulated at the wafer’s rear (unload) side. The current can be registered in the galvanic cell (Figure 10). The current flows through the electrolyte between the Pt-cathode and Si/Pd-anode. The Pt-electrode is arranged in the electrolyte in immediate proximity to the sample surface.

**Figure 12:**
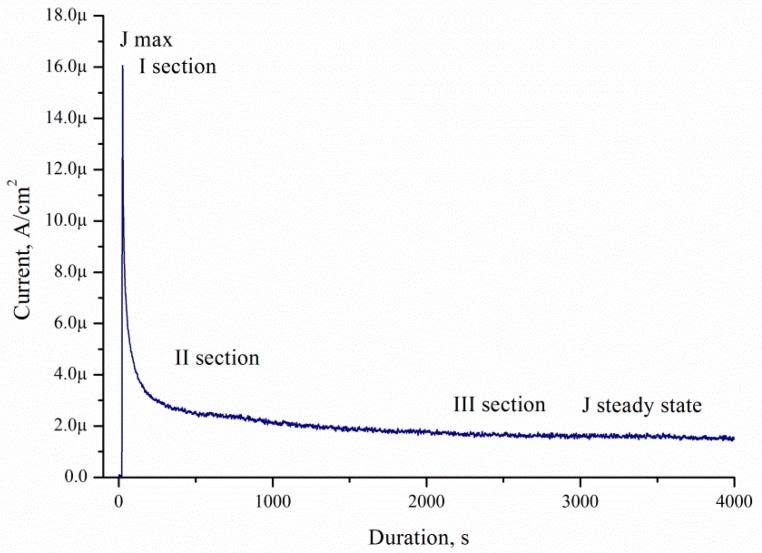
Typical *J*(*t*) sections.

The current density decreased with time for all porous samples and solutions. All catalysts showed the maximum current densities (*J*_max_) immediately after the step (I section) (Figure 12). Then, the current decreased with time (II section). After a few minutes, the current achieved a pseudo-steady state (III section). The curve type corresponds to current–time curves measured during electrooxidation of dimethyl ether on Pt/C and PtMe/C catalysts in sulphuric acid [24].

Decrease of the current density is due to the formation of the contamination on the porous silicon surface. Table 5 shows the current–time curves analysis for different solutions. 

It was established that *J*_max_ depends on the thickness of the porous silicon and concentration of ethanol. Such dependence is due to the EEO reactions yield. In this case, the thickness of the porous layer affects the amount of Pd particles in the porous layer, due to the sample preparation. The minimum value of *J*_max_ and *J*_steady state_ is observed for the case with a minimum porous layer thickness of 8 μm. The maximum values of *J*_max_ equal to 77 and 79.6 μA/cm^2^, and *J*_steady state_ equal to 7 and 5.5 μA/cm^2^, are observed for 38–40 μm thick porous layers. Ethanol concentration in solution does not affect *J*_steady state_, but affects *J*_max_. *J*_max_ reaches a value between 17 to 23 μA/cm^2^ for solution 10/90. *J*_max_ reaches a value between 33 to 79.6 μA/cm^2^ for solution 95/5. This may be due to the non-wettability of the surface of porous silicon formed Pd nanoparticles-assisted etching [25] by solution 95/5 and 50/50 (the contact angles are 140°). The contact angle for solutions 10/90 is 140°. The higher the ethanol concentration, the less the contact angle on porous surface [26]. Solution 10/90 showed the best activity for the electrooxidation in this study because of the high value of *J*_steady state_. Low surface contamination during electrooxidation (Figure 8c) facilitates ethanol access to the Pd surface, intensive mass transfer, and high *J*_steady state_ value.

**Figure 13:**
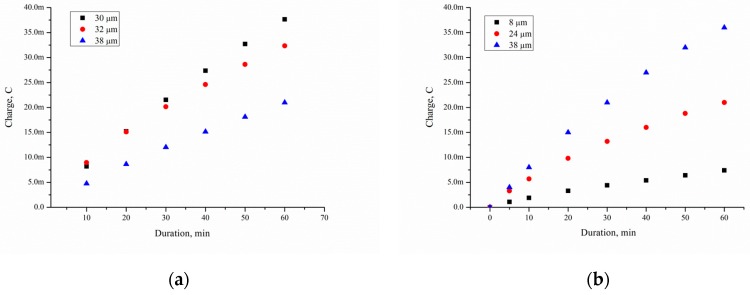

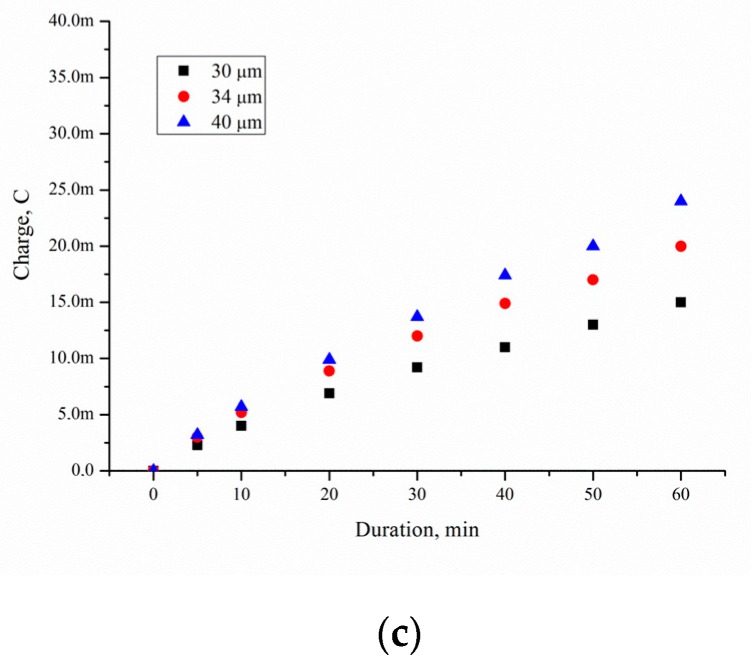
The *Q*_Excess Carrier_ versus duration of ethanol oxidation of porous silicon with different thickness and solution: (**a**) 10/90, (**b**) 50/50, (**c**) 95/5.

The corresponding charges from Figure 11 can be extracted and plotted versus a time scale to indicate the rate of formation of adsorbed species at this preparation potential of Pd/por-Si. The value of *Q*_Excess Carrier_ was determined by numerical integration of the dependence of the current on time and presented in Figure 13. The *Q*_Excess Carrier_ value is characterising excess charge carriers diffused into the substrate during EEO.

The charge of time is described by a polynomial of degree 2 with *R* = 0.99%. The charge passing through the substrate *Q*_Excess Carrier_ depends on a few factors: concentration of charge carriers injected into silicon *Q*_total_, substrate thickness and specific resistivity (*Q*_sub_), and porous layer thickness (*Q*_por-Si_).

(5) $$Q_{total}=Q_{Excess\;Carrier}+Q_{sub}+Q_{por}$$

*Q*_total_ characterises the value of all charge carriers involved in the electrooxidation of ethanol. It the case of the present research work, *Q*_total_ depends on Pd/por-Si contact area. The increase in charge carrier concentration on the surface of the hole, caused by injection, leads to the appearance of a diffusion electron flow directed along the x-axis perpendicular to the semiconductor surface, with the result that the carrier concentration increases not only on the surface but also in the depth of the semiconductor. In this case, injected carriers go deeper into the semiconductor at different distances, where they are recombined.

**Figure 14:**
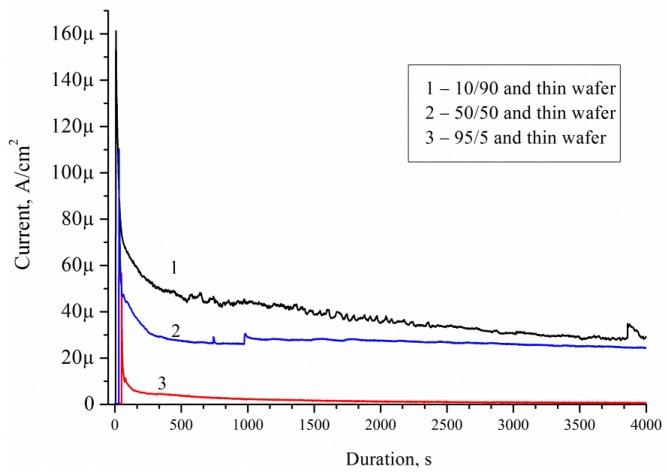
Current–time curves measured on Pd/por-Si for solutions: 10/90, 50/50 and 95/5.

Figure 14 shows current–time curves measured on Pd/por-Si for different solutions at 25 °C. A decrease in the wafer thickness by 70 μm, with the same thickness of porous silicon 30 μm, increases the charge from 36 to 140 mC for solution 10/90, from 20 to 100 mC for solution 50/50, and from 14 to 72 mC for solution 95/5 at 3600 s oxidation.

Having excluded the contribution of the thickness and resistivity of a single crystal silicon wafer, as well as the thickness of the porous layer because of equal value, we can obtain the equation:

(6) $$Q_{total\;n}-Q_{Excess\;Carrier\;n}=Q_{total\;m}-Q_{Excess\;Carrier\;m}$$

where *n* and *m* are the solutions number, and *Q*_Excess Carrier_ is measured by short-circuit current in the galvanic cells. We can calculate *Q*_total_ for unknown solutions for any duration, using the same porous silicon samples and one test solution with *Q*_total_.

**Figure 15:**
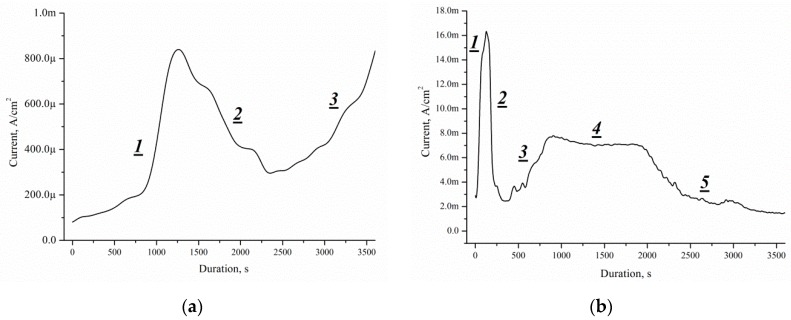
Current–time curves measured during Pd nanoparticles-assisted etching of silicon with thickness: (**a**) 525 μm, (**b**) 336 μm.

This approach can be used for porous silicon formation by Pd nanoparticles-assisted etching. The process of forming a porous layer is identical, with the only difference being that the thickness of the initial single crystal is 525 μm (Figure 15a) and 336 μm (Figure 15b), respectively.

The *J*(*t*) curves characterise the etching mechanism. Five characteristic regions can be identified:

(I) current increasing,
(II) slowing growth rate and subsequently decreased current,
(III) current increasing,
(IV) constant current,
(V) current decreasing.

*J*(*t*) reflects a change of the area (*S*) of the electrochemical reaction front. The changes of *J* with time (Figure 14) are related to the evolution of the Si morphology during pore nucleation. Pore formation takes place after the immersion of silicon into the solution containing HF and H_2_O_2_. The pore area depends on the duration of the treatment [27]. The increase in the surface area leads to the growth of the current density, the first region.

The current growth continues until a porous layer of critical thickness is formed on the Si surface. In this case, access of the solution to the surface of monocrystalline silicon becomes limited. The transport of holes through porous silicon is difficult due to the high specific resistance of the por-Si [27]. With an increase in the thickness of the porous layer, the concentration of excess holes that are not involved in the dissolution of Si becomes smaller, the second region. A further effect of the solution on the surface leads to the dissolution of the porous layer (the beginning of region III). Dissolution of por-Si reduces the thickness of the porous layer to less than the critical value, which increases the concentration of holes in Si, and, consequently, the current in region III. The current is constant in region IV due to the growth and etching of the porous silicon. The porous layer growth and current decrease in the region V.

The charge of the first cycle (single crystal is 525 μm) takes the value of 1.5 C, while the second (single crystal is 336 μm) 17.7 C. Thus, in a single crystal 189 μm thick with a specific resistance of 0.01 Ω·cm, carriers of charge of 16.2 C recombine. Removing silicon can increase the charge passing through the sample by 11.8 times. The currents of the second cycle have values exceeding the currents of the first cycle and allow a detailed study of the structural change during Pd nanoparticles-assisted chemical etching of silicon.

## 5. Conclusions

It is shown that por-Si, formed by Pd nanoparticles-assisted chemical etching, has the property of ethanol electrooxidation. Intense gas evolution is observed from the metal/porous silicon immersed in ethanol solutions. The chromatographic analysis of EEO products on por-Si/Pd shows that the main products are CO_2_, CH_4_, H_2_, CO, O_2_, methanol and water vapor. The duration of the gas evolution is linearly dependent on the porosity of the layer. Therefore, the high porosity of the sample ensures access of the reactants to the surface of por-Si/Pd and removal of the reaction products. The gradual reduction of the gas evolution rate was due to contamination of the surface with reaction products. The mass activity, as a function of time, was measured by the short-circuit current in the galvanic cells.

It was established that *J*_max_ depends on the thickness of the porous silicon and concentration of ethanol. Such dependence is due to the EEO reactions yield. In this case, the thickness of the porous layer affects the amount of Pd particles in the porous layer, due to the sample preparation. The minimum value of *J*_max_ and *J*_steady state_ is observed for the case with a minimum porous layer thickness of 8 μm. The maximum value of *J*_max_ equals to 77 and 79.6 μA/cm^2^, and for *J*_steady state_ equals to 7 and 5.5 μA/cm^2^, is observed for 38–40 μm thick porous layers. Ethanol concentration in solution does not affect *J*_steady state_, but affects *J*_max_. *J*_max_ reaches a value between 17 to 23 μA/cm^2^ for solution 10/90. *J*_max_ reaches a value between 33 to 79.6 μA/cm^2^ for solution 95/5.

A decrease in the wafer thickness by 70 μm, with the same thickness of porous silicon, increases the charge carriers, diffused into the substrate, from 36 to 140 mC for solution 10/90, from 20 to 100 mC for solution 50/50, and from 14 to 72 mC for solution 95/5 at 3600 s oxidation. Thus, the porous silicon thickness, porosity and solution composition are the main factors defined EEO efficiency.

## Figures and Tables

**Table 1:** Solution properties.

Solution	Ethanol/Water (Volume Ratio)	pH	Conductivity
1	10/90	2	5.00 mS/cm
2	50/50	2	2.84 mS/cm
3	95/5	2	0.514 mS/cm

**Table 2:** The rate of gas evolution for three ethanol solutions.

Volume Ratio C_2_H_5_OH/H_2_O	95/5	60/40	30/70
***t*, s**	120	540	105
	360	900	870
	840	2550	2760
***V*·10^−3^, cm^3^/s**	19.8	4.3	22.6
	6.6	2.6	2.6
	2.8	1	0.8

**Table 3:** The element analysis of Spot 2 for porous silicon after 120 min electrooxidation of different solutions.

Volume Ratio C_2_H_5_OH/H_2_O	Elements	Weight %	Atomic %
95/5	C	2.37	5.23
	O	3.33	5.53
	Si	94.30	89.23
60/40	C	4.82	10.16
	O	8.35	13.23
	Si	86.83	76.61
30/70	C	3.20	7.05
	O	2.56	4.23
	Si	94.23	88.71

**Table 4:** Surface bonding of porous silicon after electrooxidation (EEO).

Wave Number, cm^−1^	Bonds, Vibration Mode
625	Si–H wag [19]
1391	*δ*(C−H) of CH_3_CH_2_OH
1400	acetate νs CH_3_COO^−^ [20]
1452	*δ*(C−H) of CH_3_CH_2_OH
1550	acetate νs CH_3_COO^−^ [20]
2850	CO_3_^2−^
2900	*ν*(C−H) of CH_3_CH_2_OH [21]

**Table 5:** The current–time curves analysis for different solutions.

Volume Ratio C_2_H_5_OH/H_2_O	Porous Silicon Thickness, μm	*J*_max_, μA/cm^2^	*J*_steady state_, μA/cm^2^
10/90	30	23	8
	32	22	6
	38	17.5	5
50/50	8	16	2
	24	52.5	3.5
	38	77	7
95/5	30	54	3
	34	33	4.5
	40	79.6	5.5
